# Experimental and Numerical Investigation of Cold-Formed Steel Storage Platforms with Perforated Channel Beams

**DOI:** 10.3390/ma19153316

**Published:** 2026-08-04

**Authors:** Szymon Swierczyna

**Affiliations:** Faculty of Civil Engineering, Silesian University of Technology, Akademicka 5, 44-100 Gliwice, Poland; szymon.swierczyna@polsl.pl

**Keywords:** cold-formed steel, perforated channel sections, flexural behavior, local–distortional buckling interaction, GMNIA, web perforations, storage platforms

## Abstract

This paper presents the results of experimental investigations of the flexural capacity of the main beams in a storage platform with plan dimensions of 5.0 m × 2.0 m. The platform was designed as a grid structure, with main beams made of cold-formed channel sections and crossbeams made of sigma-section members. The crossbeams, spaced at 0.6 m, were connected to the main beams using M12 bolts and angle cleats. Eight test specimens were examined, with section heights ranging from 250 to 500 mm and wall thicknesses of 3 or 4 mm, fabricated from S350GD+Z steel. Loading was applied in a four-point bending scheme until failure of one of the main beams, while recording the moment–deflection relationship. The obtained failure loads were compared with the design resistances calculated in accordance with EN 1993-1-3. Additionally, GMNIA analyses were performed for the tested storage-platform structures using the Idea StatiCa Member version 24.1 software, incorporating measured material properties and equivalent geometric imperfections in accordance with prEN 1993-1-14. The adopted procedure included a sensitivity study to investigate the influence of different combinations of local and distortional buckling mode imperfections on the numerical results. The observed behaviour was characterized by interaction between distortional and local buckling modes, accompanied by yielding in the compression zone. The GMNIA analyses predicted the ultimate bending resistance with good accuracy, yielding FEM-to-test resistance ratios between 0.93 and 0.98. The sensitivity study indicated that the predicted ultimate resistance was only weakly affected by the assumed combination of local and distortional imperfection modes. For the investigated platform systems, the effective section approach according to Eurocode 3 provided accurate predictions of the ultimate resistance, with calculated-to-test resistance ratios ranging from 0.96 to 1.01. This agreement is discussed in the context of the possible stiffening effect of crossbeam-to-web connections.

## 1. Introduction

**Figure 1:**
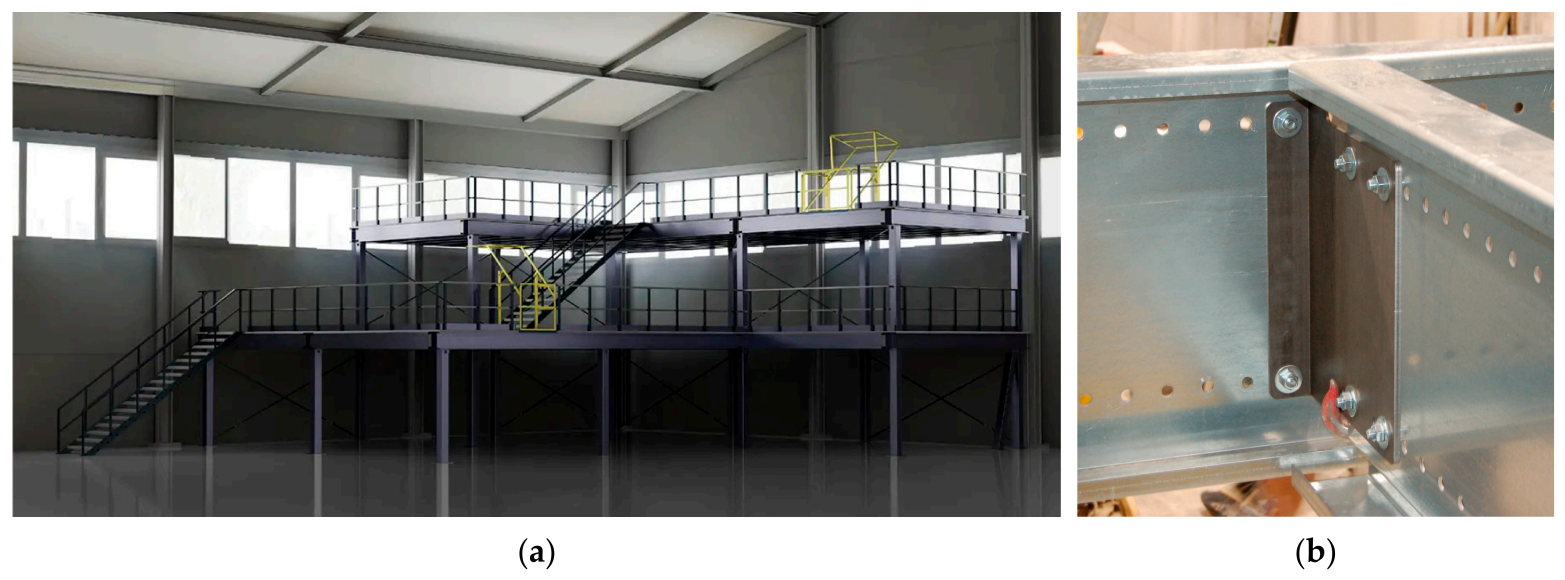
Platform made of cold-formed steel members: (**a**) visualization of a typical storage platform; (**b**) detail of the main-beam–crossbeam angle-cleat connection.

Existing industrial and warehouse buildings can significantly increase their usable floor area through the installation of additional intermediate floors, commonly referred to as mezzanines. Such structures should be capable of carrying substantial loads while remaining lightweight and easy to transport and assemble. In addition, modular systems should be adaptable to existing conditions and allow for future modification and expansion. These requirements can be satisfied by structures made of cold-formed steel (CFS) members [1,2], for example in the form of platforms supported by appropriately braced steel columns (Figure 1a). Such platforms typically consist of main beams and crossbeams fabricated from cold-formed channels or sigma sections connected by means of angle cleats and bolts (Figure 1b). The platform decking may be formed, for example, from composite panels or steel bar grating.

The optimum design of cold-formed steel structures requires consideration of complex local, distortional and global stability phenomena [3]. It should also be noted that the walls of such members frequently contain various types of openings and perforations, which facilitate the installation of services, reduce the self-weight of the structure, and provide locations for mechanical fasteners (Figure 1b).

Moen and Schafer [4] investigated the influence of openings on the elastic critical stresses associated with the local buckling of plates subjected to compression or bending. In a subsequent study [5], these investigations were extended to include the global (flexural and lateral-torsional) and distortional buckling of cold-formed steel members with oval web openings of various sizes and locations. For cold-formed steel beams, the elastic lateral-torsional buckling moment decreased by only about 1% for *h_hole_*/*H* = 0.10, but by approximately 32% for *h_hole_*/*H* = 0.90, where *h_hole_* is the hole height, and *H* is the total web depth. Simplified design expressions were also proposed to account for the influence of web openings on the buckling behaviour of cold-formed steel members.

Yuan et al. [6] examined distortional buckling of cold-formed channel beams with circular web openings. Finite Element Method (FEM) results were compared with the analytical model proposed in EN 1993-1-3 [7], leading to suggested modifications of the design provisions. It was shown that increasing the hole diameter progressively reduced the distortional buckling resistance; for the largest analysed openings (*h_hole_*/*H* = 0.70), the critical distortional buckling moment decreased by approximately 22% compared with beams without web openings.

Zhao et al. [8] and Oruç et al. [9] conducted experimental and numerical investigations of cold-formed steel beams with rectangular web openings. The results demonstrated that web openings alter stress distribution and promote a transition from purely local or purely distortional buckling to an interactive local–distortional buckling mode. Zhao et al. [8] found that the moment capacity decreased by up to 7.0% for *h_hole_*/*H* ≤ 0.40 and by up to 16.3% for *h_hole_*/*H* = 0.80. More recent studies [9] further confirmed that the reduction in flexural resistance becomes increasingly pronounced for larger web openings and proposed improved design equations for local buckling.

Previous studies have demonstrated that web perforations may significantly affect the stiffness, buckling behaviour and resistance of cold-formed steel members. However, most of the available research has focused on isolated beams or columns containing relatively large openings, whose dimensions were comparable to the cross-sectional dimensions of the members. Limited information is available on members containing a dense arrangement of relatively small perforations, which are commonly used in storage-platform systems for fastening connections and accessories. In such structures, the spacing between adjacent perforations may be considerably smaller than the member depth, resulting in structural behaviour that differs from that observed in previously investigated cases.

Karmazínová et al. [10] and Horáček et al. [11] examined the effects of web openings on the lateral-torsional buckling resistance of cold-formed sigma-section beams. Based on the obtained results, a method for determining the lateral-torsional buckling resistance was proposed using the geometric properties of an equivalent cross-section defined as a weighted average of the gross section and the perforated section. In a paper by Bukasa and Dundu [12], the lateral-torsional buckling resistance of sigma-section beams was investigated experimentally. The beams were restrained against torsion by means of angle-cleat connections to channel-section purlins, which effectively reduced the buckling length to the spacing between adjacent restraints. These findings suggest that similar restraint conditions may also influence local and distortional buckling behaviour. In the storage-platform systems, where the transverse members are connected directly to the webs of the main beams, such effects may partially compensate for the adverse influence of web perforations. However, this hypothesis has received little attention in the available literature.

While the behaviour of isolated cold-formed steel members has been extensively investigated, the structural response of complete storage platform systems has received considerably less attention. In particular, there is a lack of experimental and numerical studies addressing platforms composed of perforated channel-section primary beams connected to transverse members, where the interaction between local and distortional buckling, web perforations and connection restraints governs the global structural response.

Horaček et al. [13] conducted full-scale tests on lightweight steel-framed floor systems composed of cold-formed lipped channel beams and joists connected by angle cleats. Their research focused on the fire resistance of unprotected floor systems and the applicability of Eurocode fire design provisions. Although the study provided valuable information on the behaviour of complete structural assemblies, it was limited to elevated-temperature conditions and did not address the structural response, stability behaviour or load-carrying resistance of storage platform systems under ambient conditions.

Consequently, the applicability of conclusions drawn from tests on isolated members to complete storage platform systems remains uncertain. In practical structures, the structural response is influenced not only by the behaviour of individual perforated beams but also by their interaction with transverse members, connection details and the restraint provided by adjacent structural components. These effects may significantly modify the development of local and distortional buckling and, in turn, the ultimate load-carrying resistance of the system.

To address these limitations, the present study combines full-scale experimental testing with validated GMNIA of complete storage-platform systems incorporating perforated cold-formed steel channel beams. The proposed approach enables the behaviour of complete structural systems to be investigated rather than isolated members. Unlike previous investigations focused on isolated members, the study captures the interaction between perforated primary beams, transverse members and their connections, enabling the influence of these interactions on local–distortional buckling and structural resistance to be assessed. In addition to evaluating the accuracy of resistance predictions obtained using the Eurocode effective width method, the study provides new insight into the structural behaviour of practical storage-platform systems and the mechanisms governing their flexural resistance.

Owing to the high slenderness of cold-formed steel members, their structural behaviour is highly sensitive to geometric imperfections and residual stresses induced during the manufacturing process [14]. Previous studies have consistently shown that geometric imperfections generally exert a more pronounced influence on the behaviour and resistance of cold-formed steel members than residual stresses. Consequently, accurate representation of their shape, distribution and magnitude is essential for reliable numerical simulations [15,16,17,18]. In advanced finite element analyses, geometric imperfections are commonly introduced by scaling selected buckling modes obtained from a Linear Buckling Analysis (LBA), providing the basis for Geometrically and Materially Nonlinear Analysis with Imperfections (GMNIA) [17,18].

Procedures for determining the ultimate resistance of steel structures using GMNIA are provided in prEN 1993-1-14 [19]. The standard recommends the use of equivalent geometric imperfections that account for the combined effects of geometric imperfections, residual stresses and modelling uncertainties. For cold-formed steel members, particular attention should be paid to the selection of representative combinations of local, distortional and global imperfections. In practice, however, eigenmodes obtained from LBA frequently contain several interacting instability components, making the identification of the most critical imperfection pattern a challenging task.

Accordingly, the present study also examines the influence of different local–distortional geometric imperfection patterns on the predicted resistance of storage-platform systems, thereby assessing the robustness of the adopted GMNIA modelling approach.

## 2. Materials and Methods

### 2.1. Experimental Study

Unlike most previous studies, the tests were performed on complete platform systems rather than isolated beams in order to account for the influence of crossbeam-to-web connections on the structural behaviour of the main members. The main beams were fabricated from cold-formed lipped channel sections C250 × 3, C250 × 4, C400 × 3, C400 × 4, C450 × 3, C500 × 3 and C500 × 4, while the corresponding crossbeams were fabricated from sigma sections S250 × 3, S250 × 4, S400 × 3, S400 × 4, S450 × 3, S500 × 3 and S500 × 4, respectively. 

**Figure 2:**
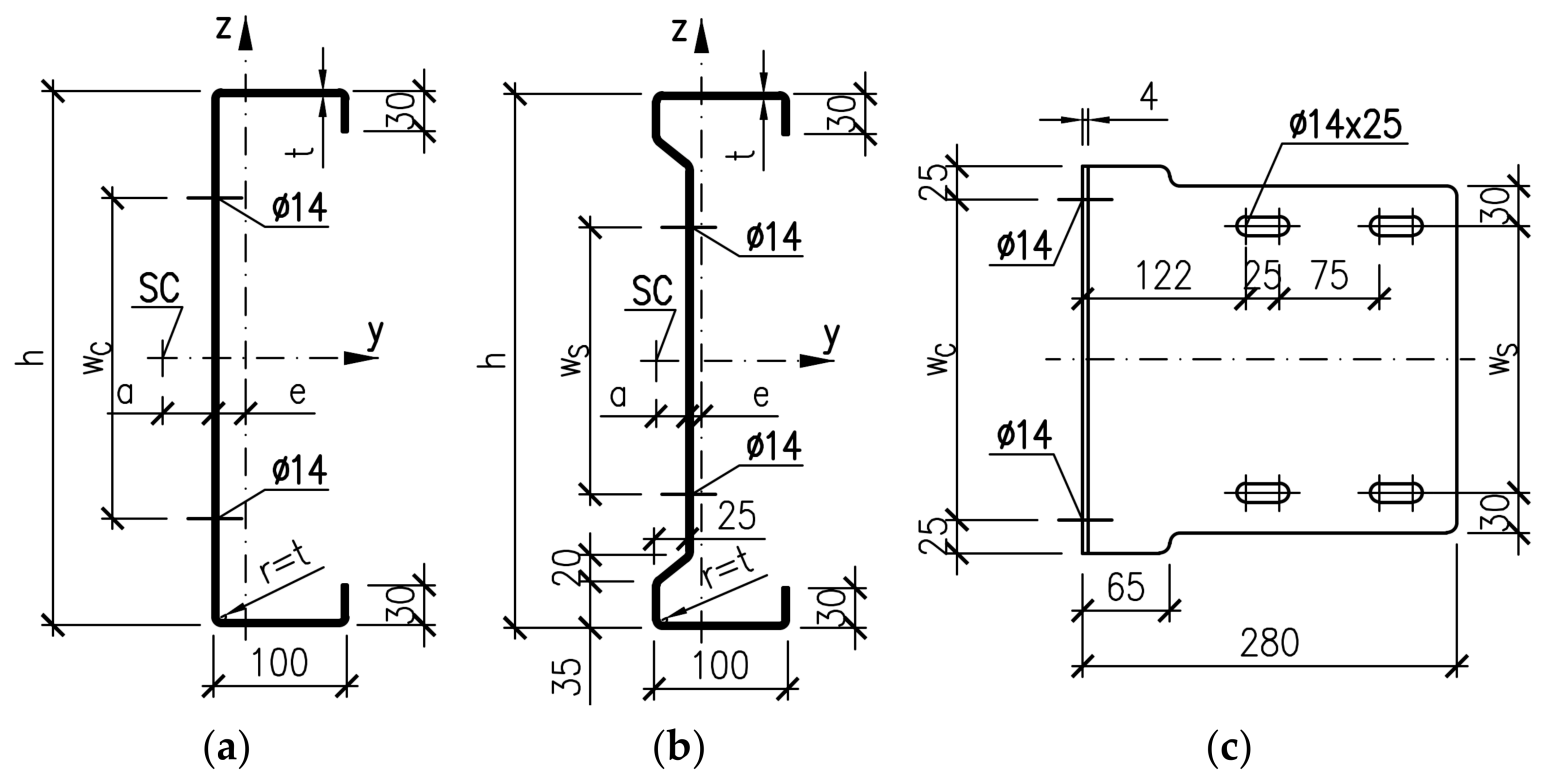
Dimensions (in millimeters) of platform cross-sections: (**a**) main beams; (**b**) crossbeams; (**c**) angle cleats, where SC denotes the shear centre and w_c_ and w_s_ denote the bolt hole spacing.

The nominal dimensions of the cross-sections are shown in Figure 2 and listed in Table 1.

The main beams were manufactured by press-braking from S350GD+Z275 steel sheets supplied in two production batches corresponding to the nominal thicknesses of 3 mm and 4 mm. All members of a given thickness were fabricated from the same material batch. The manufacturer did not specify the dimensional tolerances with reference to any execution standard applicable to press-braked sections. Therefore, where appropriate, the measured geometrical deviations were evaluated with reference to the tolerances specified in EN 10162 [20], although this standard formally applies to cold roll-formed steel sections.

Prior to cold-forming, material coupons were extracted from the steel sheets and subjected to standard tensile coupon tests. The obtained material properties are summarized in Table 2.

**Figure 3:**
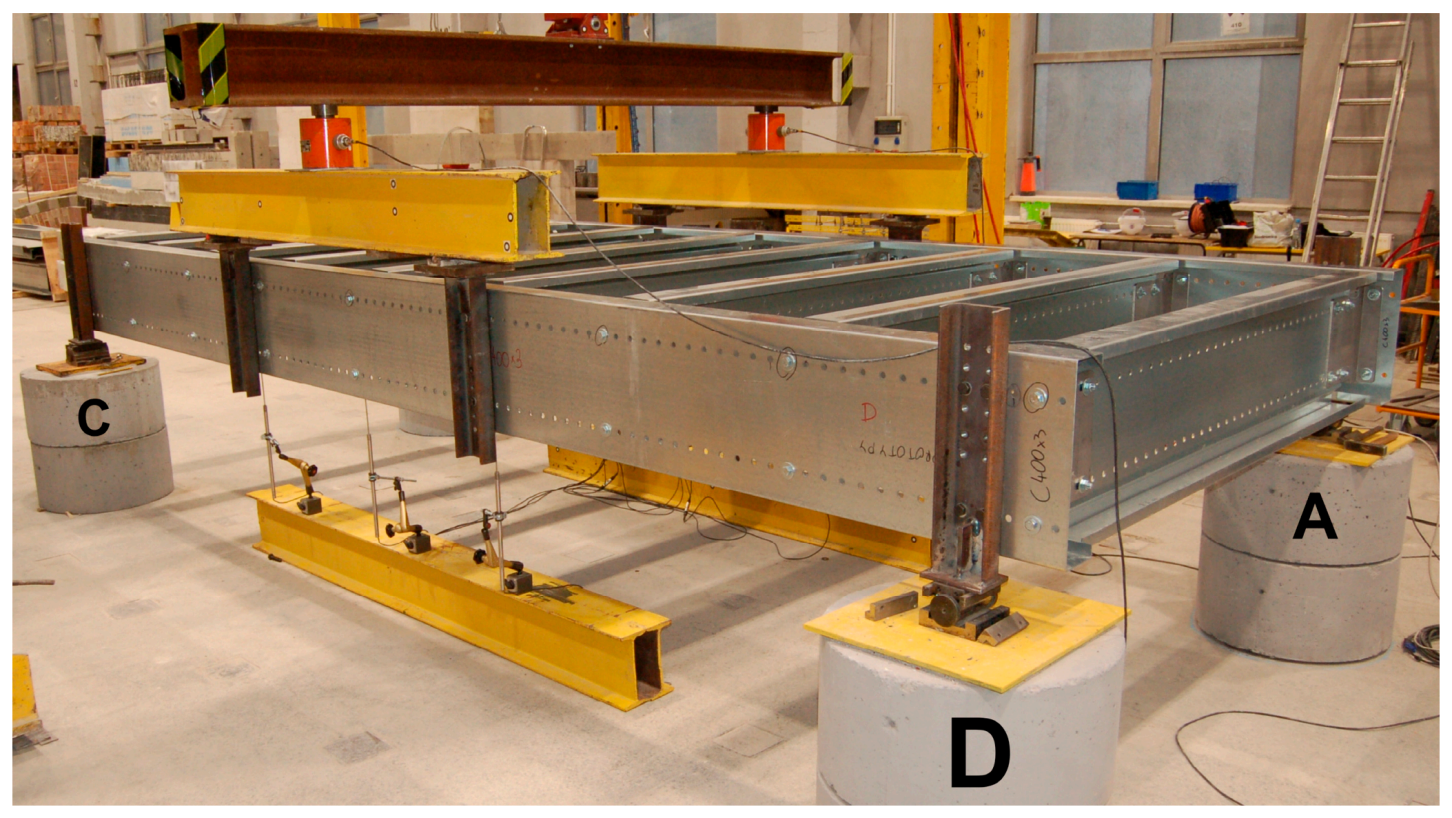
View of the test stand, where A, C and D denote the supports.

Two rows of Ø14 mm holes were provided in both the main beams and the crossbeams at a longitudinal spacing of 50 mm. These holes were used to connect the members by means of S235 steel angle cleats (see Figure 2c) and M12 grade 8.8 bolts. Each platform, with an overall width of 2.0 m, consisted of nine crossbeams spaced at 600 mm centres. A view of a test specimen mounted on the test stand is shown in Figure 3.

One platform was tested for each main beam size, with the exception of C250 × 3, for which two identical specimens, designated C250 × 3.1 and C250 × 3.2, were examined.

**Figure 4:**
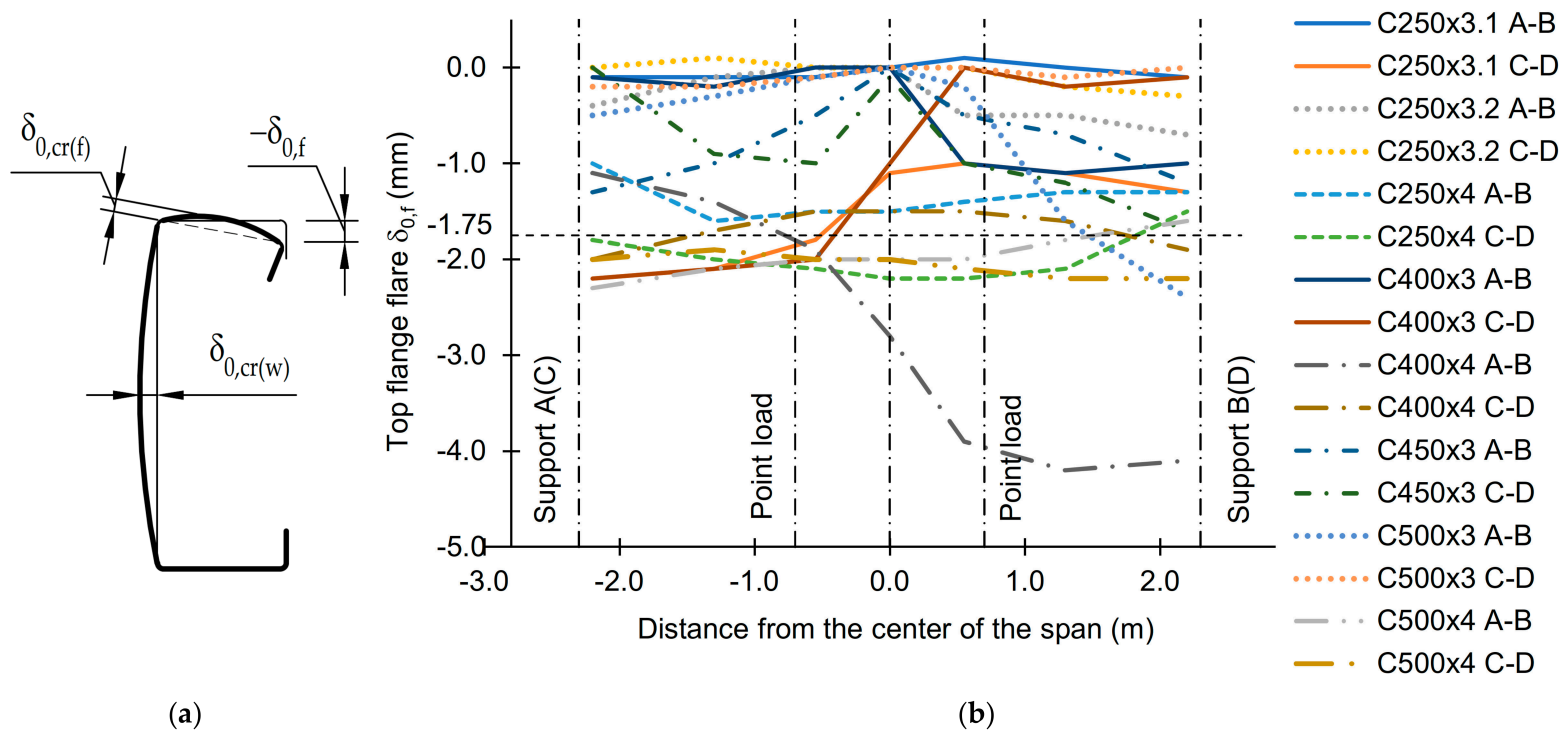
Initial geometric imperfections of the main beams. (**a**) Definition of local geometric imperfections. (**b**) Measured top flange flare imperfection of the main beams.

The geometric measurements were carried out immediately before testing, after the specimens had been positioned in the test setup. This ensured that the recorded geometry corresponded to the actual configuration at the beginning of the loading test. Cross-sectional dimensions were verified at both ends of each main beam using a digital caliper and a micrometer screw gauge with an accuracy of 0.01 mm. Geometric imperfections were then determined using straightedges, precision engineer’s squares, and wedge and leaf feeler gauges with a measurement accuracy of 0.1 mm. The web crown, flange crown and top flange flare imperfections were determined at seven locations along the beam length (Figure 4), namely at mid-span and at distances of ±0.55 m, ±1.30 m and ±2.20 m from the beam centre. The global bow imperfection was measured at mid-span relative to a chord connecting the corners of the top flanges of the channel section.

It was found that the measured plate thicknesses and widths differed only marginally from their nominal values. Table 3 summarizes the maximum recorded values of the cross-sectional imperfections in the individual main beams, as defined in Figure 4a. The main beams were designated A–B and C–D according to the support labels (see Figure 3).

The above imperfections were found to be slightly smaller than the statistically derived mean values reported by Zeinoddini and Schafer (2012) [17]. In contrast, the top flange flare imperfection was found to be significant. Its variation along the length of the main beams is illustrated in Figure 4b.

In the vast majority of cases, the imperfection was directed towards the bottom flange, indicating that it originated during press-brake forming as a result of overbending. In some beams, however, the deformation exhibited an irregular pattern, suggesting that additional distortions may have occurred during storage and transportation of the platforms. In several specimens, the measured flange flare exceeded the fabrication tolerances specified in EN 10162 [20]. The largest value, *δ*_0,*f*_ = −4.2 mm, was recorded for beam C400 × 4A–B, whereas the permissible fabrication tolerance according to [20] is ±1.75 mm.

**Figure 5:**
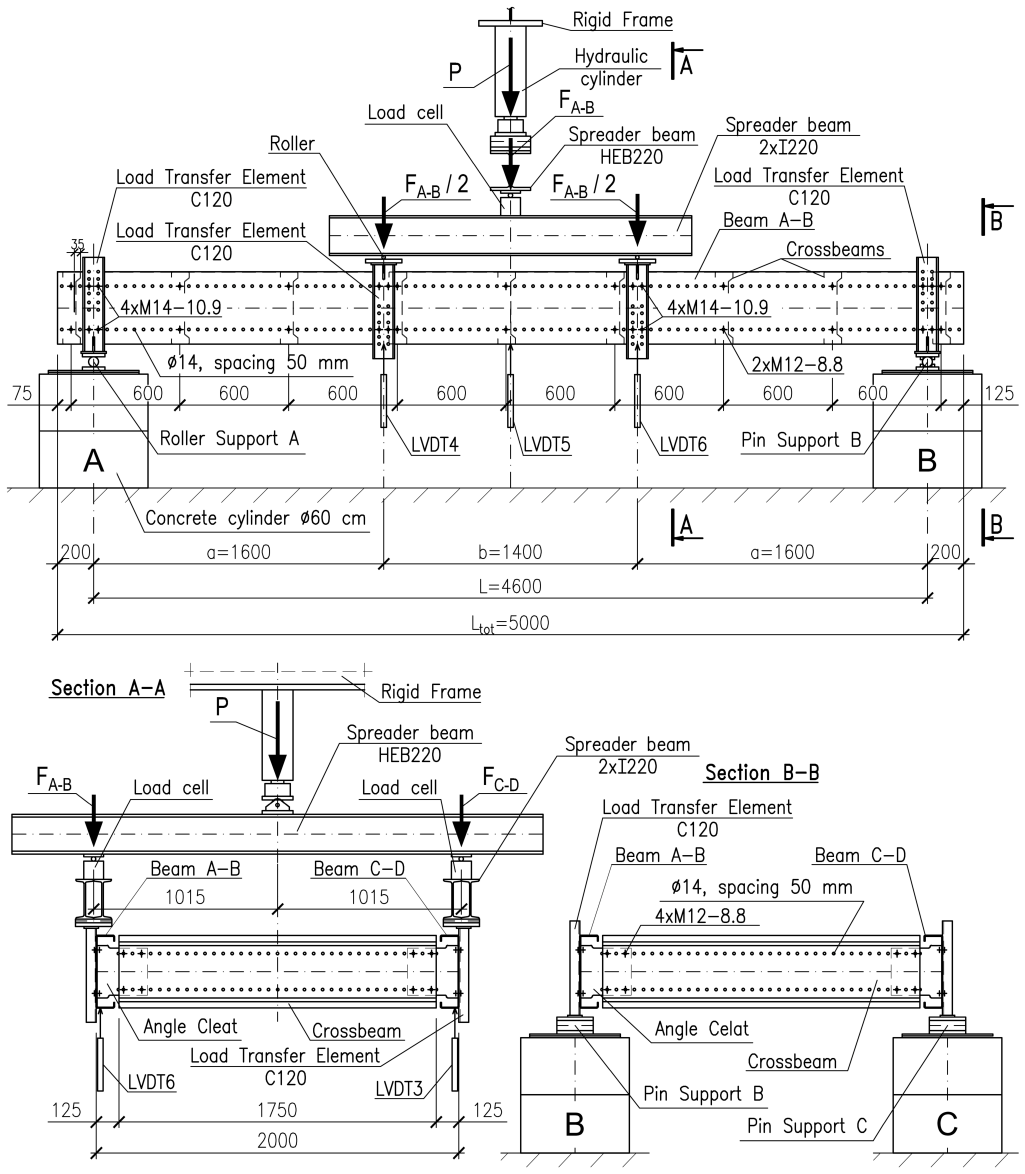
Test setup.

The test setup is shown in Figure 5. The tests were carried out under a four-point bending configuration with a main beam span of *L* = 4.6 m. The load *P* was applied by a double-acting hydraulic cylinder D10032 (Larzep Hydraulic, Mallabia, Spain) with a nominal capacity of 1000 kN positioned above the geometric centre of the platform and subsequently distributed to the two main beams by means of a spreader beam fabricated from an HEB220 section. A second spreader beam, consisting of two I220 sections, was placed on each main beam and transferred the load symmetrically at two points spaced *b* = 1.4 m apart and located *a* = 1.60 m from the supports (see Figure 5). At the supports and load application points, special load transfer elements fabricated from hot-rolled C120 channel sections were attached to the webs of the main beams using four M14 grade 10.9 bolts installed through the existing holes. The load transfer elements were introduced to prevent local crushing and to distribute concentrated forces over a larger portion of the web.

Friction at the supports was not considered in either the experimental interpretation or the numerical model. At supports A and D (Figure 5), roller supports were used to allow free longitudinal displacement of the main beams. At supports B and C, longitudinal movement was restrained by locking the rollers in the beam axis direction.

No rotational restraint was intentionally provided at any of the supports. A small rotational stiffness about the longitudinal axis of the main beams may have resulted from the contact between the 80 mm wide rollers and the steel end plates welded perpendicular to the ends of the load transfer elements. However, this effect was considered negligible. Accordingly, the finite element model assumed idealized support conditions with free rotations about all three axes.

During the tests, the applied load *P* was increased monotonically, at a rate ranging from approximately 20 kN/min in the initial stage of loading to about 1 kN/min immediately prior to failure of one of the main beams, which was indicated by a sudden increase in deflection. Two CT50 load cells with a measuring range of 500 kN installed between the HEB220 and 2 × I220 spreader beams were used to measure the forces (*F_C__–__D_*) and (*F_A__–__B_*) transmitted to the individual main beams. The deflection of each main beam was recorded using LVDT100 (Peltron, Wiązowna, Poland) displacement transducers (100 mm measuring range, 0.01 mm resolution) positioned at mid-span (LVDT5 beneath beam A–B and LVDT2 beneath beam C–D) and at the load application points (LVDT4 and LVDT6 beneath beam A–B, and LVDT1 and LVDT3 beneath beam C–D; see Figure 5). The measurement signals were acquired using a QuantumX MX840B data acquisition system (HBM, Darmstadt, Germany) and recorded with the dedicated Catman software, version 5.6.2. The ultimate bending moment was calculated separately for each main beam using the force measured by the corresponding load cell. The reported ultimate resistance corresponds to the beam that failed first. The tests were documented by digital photographs taken using a Nikon D50 camera (Nikon Corporation, Tokyo, Japan).

### 2.2. Numerical Analysis

The numerical model was developed in IDEA StatiCa Member, version 24.1 (IDEA StatiCa, Brno, Czech Republic) [21]. The program implements the Component-Based Finite Element Method (CBFEM), in which structural members are represented by shell elements and bolted connections by nonlinear spring components [22,23]. The walls of the cold-formed members were modelled using four-node quadrilateral shell elements with six degrees of freedom [22].

Contact between the angle cleats and the connected beam webs was modelled using a nonlinear one-sided contact formulation. Compression between the contacting surfaces was transferred through a penalty-based contact algorithm, while separation of the plates was allowed. Consequently, the connected plates were not assumed to be perfectly bonded.

Bolts were represented by dependent nonlinear springs whose constitutive relationships account for the behaviour of the fasteners in tension, shear and bearing [23]. The stiffness of the individual connection components was determined automatically from the connection geometry, bolt properties and material characteristics in accordance with the procedures of EN 1993-1-8 [23]. The adopted modelling approach accounts for the nonlinear bearing behaviour of bolted connections, whose deformation mechanisms in cold-formed steel members have been discussed in previous experimental studies [24].

**Figure 6:**
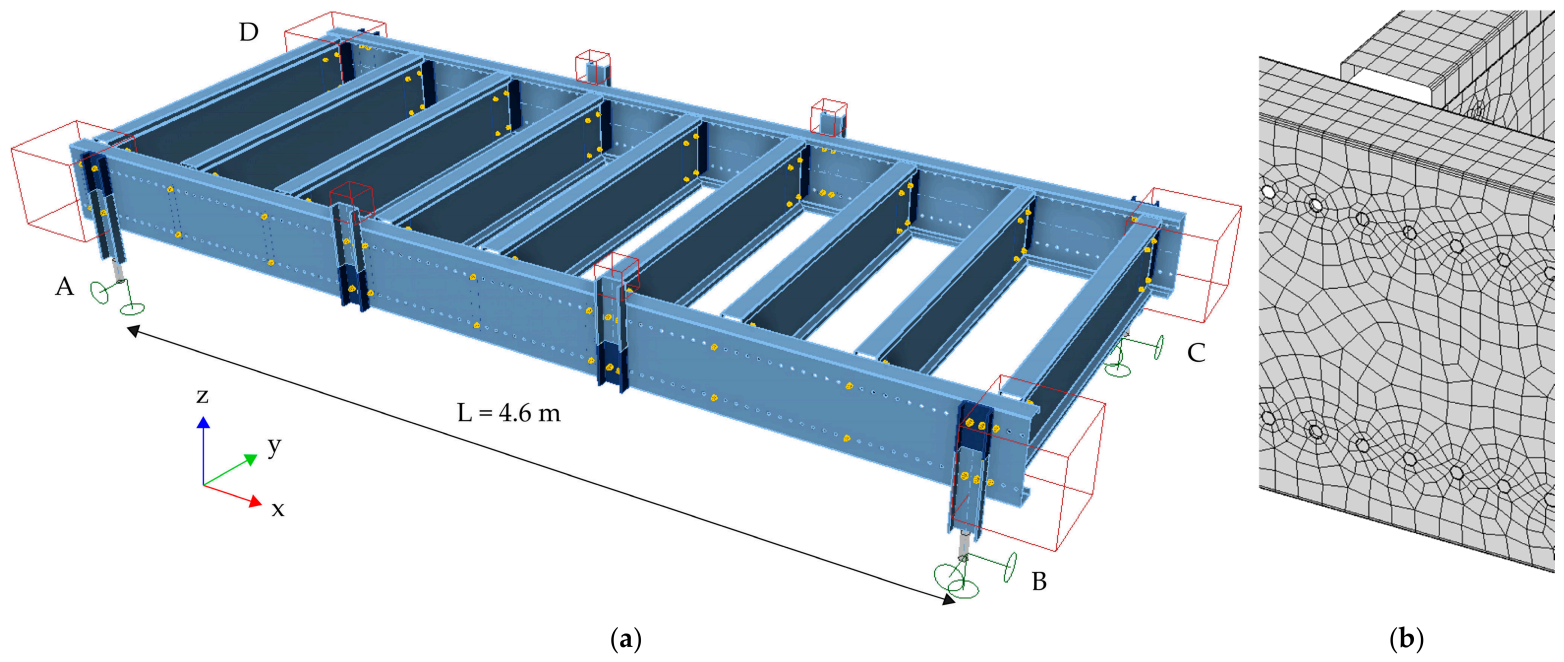
FE model of the platform: (**a**) view of the model; (**b**) details of the FE mesh.

The model included the main beams, crossbeams, angle cleats, bolted connections and load transfer elements (Figure 6a), to which the concentrated loads were applied. The self-weight of the platform members and supporting elements was taken into account as a uniformly distributed load acting along the centrelines of the main beams.

The maximum element size in the models was 20 mm. A mesh sensitivity study showed that reducing the maximum element size from 20 mm to 10 mm changed the first critical buckling load factor by approximately 2%; therefore, the coarser mesh was considered sufficient for identifying the relevant buckling modes. A comparable maximum shell-element size of approximately 20 mm was adopted in a recent CBFEM study involving LBA and GMNIA analyses performed in IDEA StatiCa Member [25]. The adequacy of the adopted discretization for the nonlinear analyses was additionally assessed through comparison of the predicted ultimate resistance, deformation patterns and failure locations with the experimental results presented later in this paper. The rounded corners of the cold-formed members were discretized using three finite elements.

The analysis procedure implemented in Idea StatiCa Member consists of three consecutive steps. The first step involves a Materially Nonlinear Analysis (MNA). The second step comprises a Linear Buckling Analysis (LBA), which is used to determine the eigenvectors *φ_i_* representing the individual buckling modes and the corresponding critical load factors *α_cr,i_*. In the third step, a Geometrically and Materially Nonlinear Analysis with Imperfections (GMNIA) is performed, in which geometric imperfections are introduced by appropriately scaling the buckling mode shapes obtained from the LBA.

Residual stresses were neither measured experimentally nor introduced explicitly into the finite element models. Likewise, although the initial geometric imperfections of the tested beams were measured experimentally, they were not introduced directly into the GMNIA models. Instead, the numerical analyses followed the procedure specified in prEN 1993-1-14 [19], in which equivalent geometric imperfections are intended to account collectively for the effects of geometric imperfections, residual stresses and modelling uncertainties. Consequently, the adopted GMNIA models do not explicitly distinguish between these individual sources of structural imperfections.

The imperfection amplitudes *e*_0_ (mm) were determined in accordance with prEN 1993-1-14 [19]. For local buckling, the following expression was adopted:

(1) $$e_{0,loc}=min\left[a/200,b/200\right],$$

where *a* (mm) and *b* (mm) denote the width and length, respectively, of the web or flange panel considered in the buckling mode.

The amplitude *e*_0,*dist*_ of the distortional imperfection was determined according to Equation (5.17) in ref. [19] as:

(2) $$e_{0,dist}=0.3\cdot t\cdot \sqrt{\frac{f_{y,b}}{\sigma_{cr,d}}},$$

where *t* (mm) is the thickness of the sheet, *f_yb_* (MPa) is the yield stress according to Table 2, and *σ_cr,d_* (MPa) is the critical distortional buckling stress. The critical distortional buckling stress was calculated as the elastic bending stress at mid-span obtained from linear analysis multiplied by the corresponding distortional buckling load factor α*_cr,dist_*.

**Figure 7:**
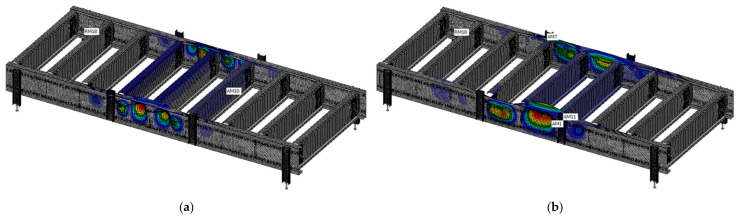
Eigenmodes used to define geometric imperfections in the GMNIA analysis of the C450 × 3 platform: (**a**) local buckling mode (α*_cr_* = 0.75); (**b**) distortional buckling mode (α*_cr_* = 0.96).

The first thirty eigenmodes were therefore classified according to their dominant instability mechanism (Figure 7). The analysed members exhibited very closely spaced eigenvalues; for example, for specimen C450 × 3, the ratio between the highest and lowest critical load factors within this group was only α*_cr,max_*/α*_cr,min_* = 1.49. When several eigenmodes exhibited similar deformation patterns and comparable critical load factors, additional GMNIA analyses were performed to identify the imperfection pattern leading to the lowest ultimate resistance.

Global bow imperfections were not included in the GMNIA models. Linear buckling analyses showed that none of the first thirty eigenmodes corresponded to a global flexural or lateral–torsional buckling mode, indicating that global instability was not governing the structural response of the tested platforms. This observation was confirmed experimentally, as no lateral–torsional buckling or significant out-of-plane displacements were observed during the tests. Moreover, the loading arrangement, in which the hydraulic actuator was connected to a stiff reaction frame, provided effective restraint against horizontal displacements perpendicular to the bending plane. The measured global bow imperfections were also very small, ranging from 0 to 1 mm (Table 3), and were therefore considered to have a negligible influence on the structural behaviour.

**Figure 8:**
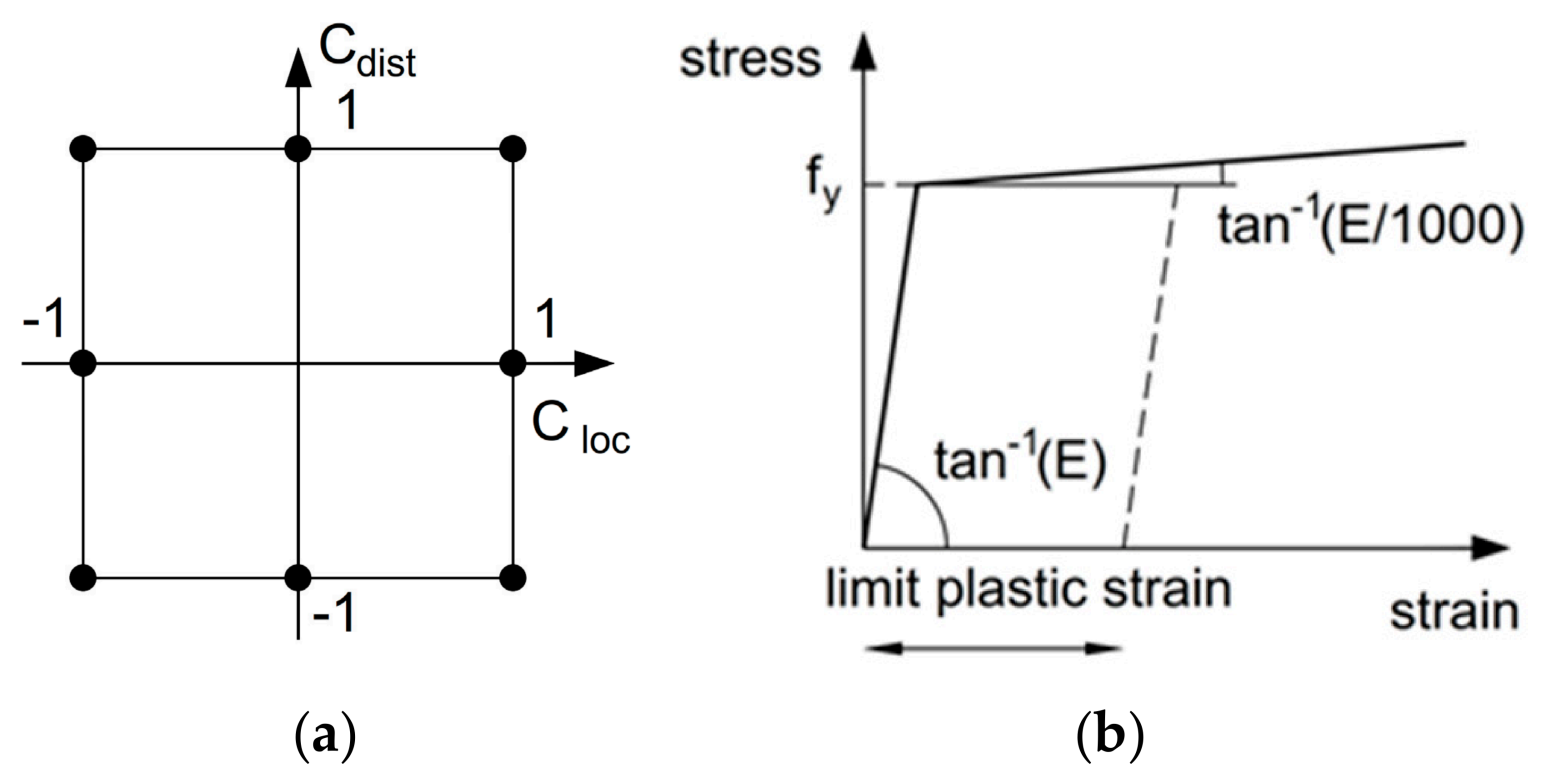
Details of the GMNIA analysis. (**a**) Square-max approach to selecting the amplitude when combining local (*C_loc_*) and distortional (*C_dist_*) imperfections. (**b**) Material model used in MNA and GMNIA analyses.

According to ref. [19], all relevant combinations of imperfections should be considered. Consequently, only local and distortional buckling modes were considered when generating geometric imperfection patterns, whereas patterns including a third global mode were not investigated. To this end, the approach proposed in ref. [17] was adopted, in which the imperfection pattern *f*_0_ is defined as:

(3) $$f_{0}=\sum_{i}e_{0,i}\cdot C_{i}\cdot \varphi_{i},$$

where *i* is the *i*-th mode shape, *e*_0,*i*_ is the amplitude of each mode, *C_i_* is a coefficient to control the sign or portion of the mode imperfection that is input, and *φ_i_* is the mode shape. To determine the most unfavourable imperfection combination, a linear combination (square-max) method was used. For each numerical model, eight geometric imperfection patterns were considered. One local buckling mode and one distortional buckling mode were scaled using the $C_{loc}$ and $C_{dist}$ coefficients, taking the values −1, 0, or +1. The resulting set comprised four single-mode patterns and four combined-mode patterns with different sign combinations, as illustrated in Figure 8a. In addition, one analysis was performed for the ideal geometry without imperfections ($C_{loc}=C_{dist}=0$).

A bilinear elastic-plastic steel model in accordance with [19] was used with the slope of the plastic branch *E*/1000 (Figure 8b). For the main beams and crossbeams, the measured yield stress *f_y_* was adopted (see Table 2), whereas Young’s modulus was taken as *E* = 210 GPa. For angle cleats and load transfer elements, the nominal mechanical properties of S235 steel, as specified in ref. [26], were adopted. The behavior of the material was based on the von Mises criterion of plasticity. The analysis was carried out until either stability collapse occurred or a predefined strain limit was reached. In accordance with EN 1993-1-3 [7], a maximum equivalent plastic strain of 5% was adopted as the limiting criterion in the GMNIA analyses.

## 3. Results

**Figure 9:**
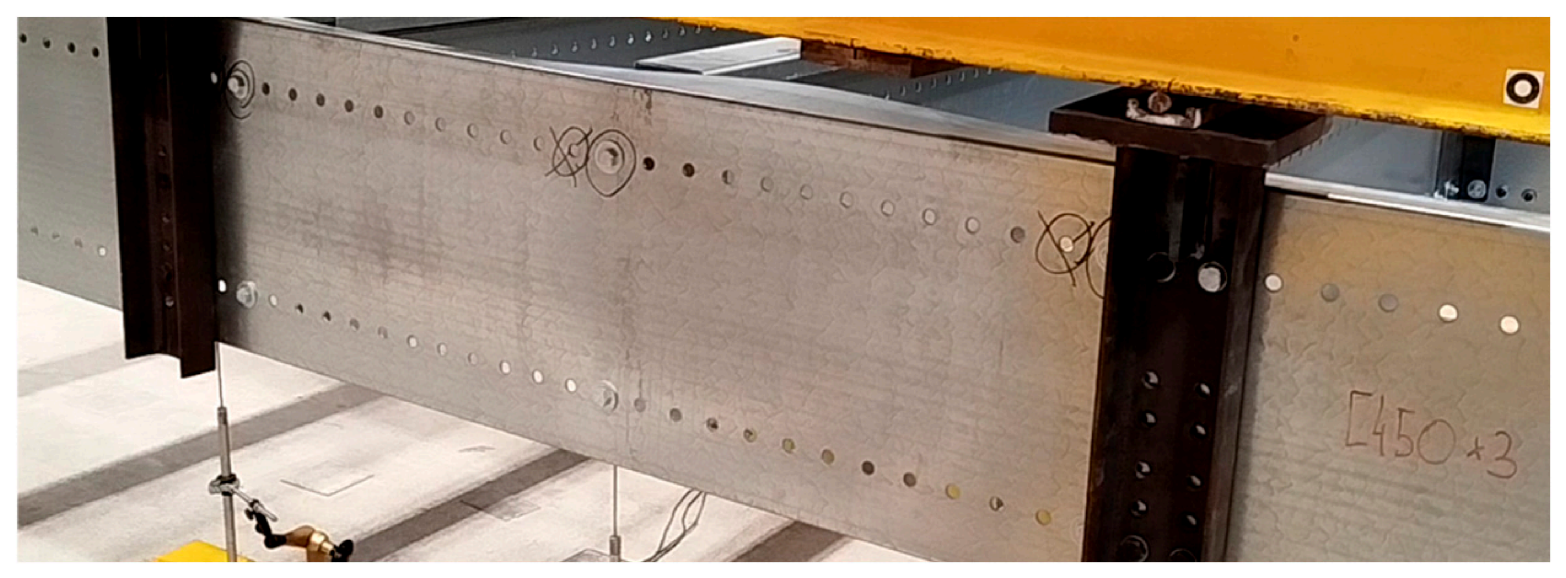
Pre-failure local–distortional deformation of the C450 × 3 platform.

Figure 9 illustrates the deformation of the main beams immediately prior to failure using specimen C450 × 3 as an example. Rotation of the compression flange, accompanied by deformation of the flange-to-web junction and local web bulging, indicates the development of distortional buckling. In all tested specimens, failure initiated in the compression zone of the web.

**Figure 10:**
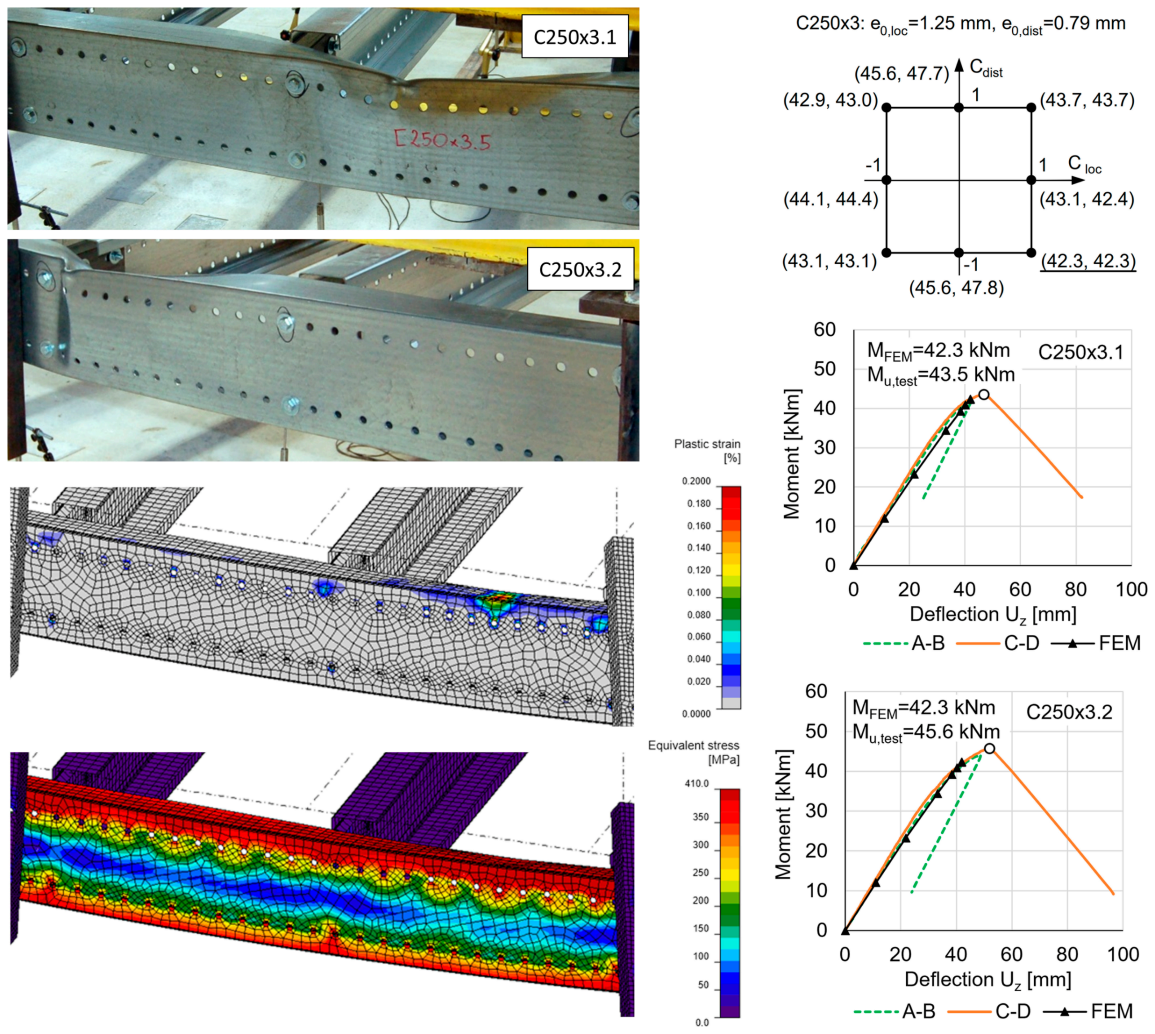
Experimental and numerical results for the C250 × 3 platform specimen.

**Figure 11:**
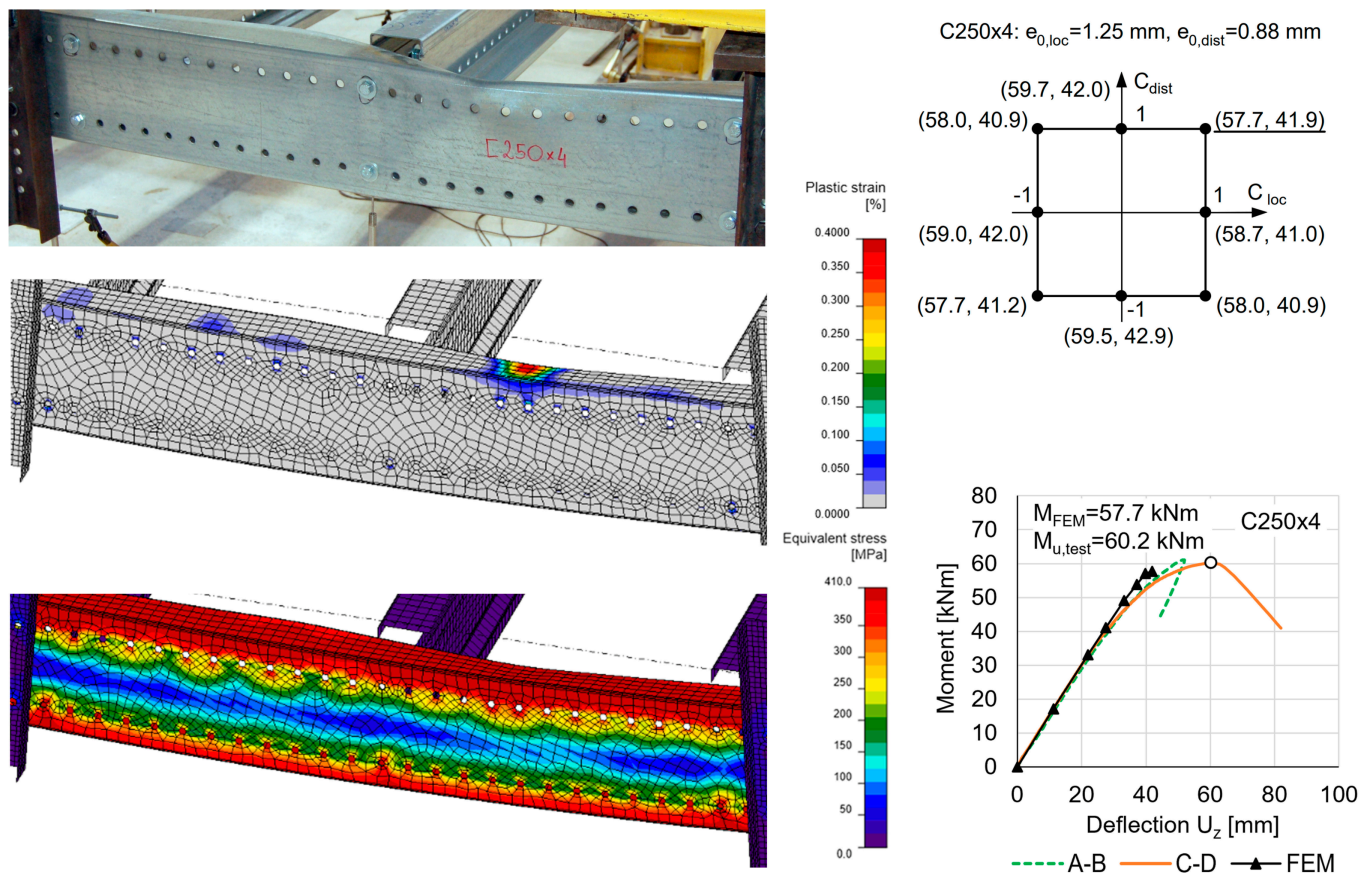
Experimental and numerical results for the C250 × 4 platform specimen.

**Figure 12:**
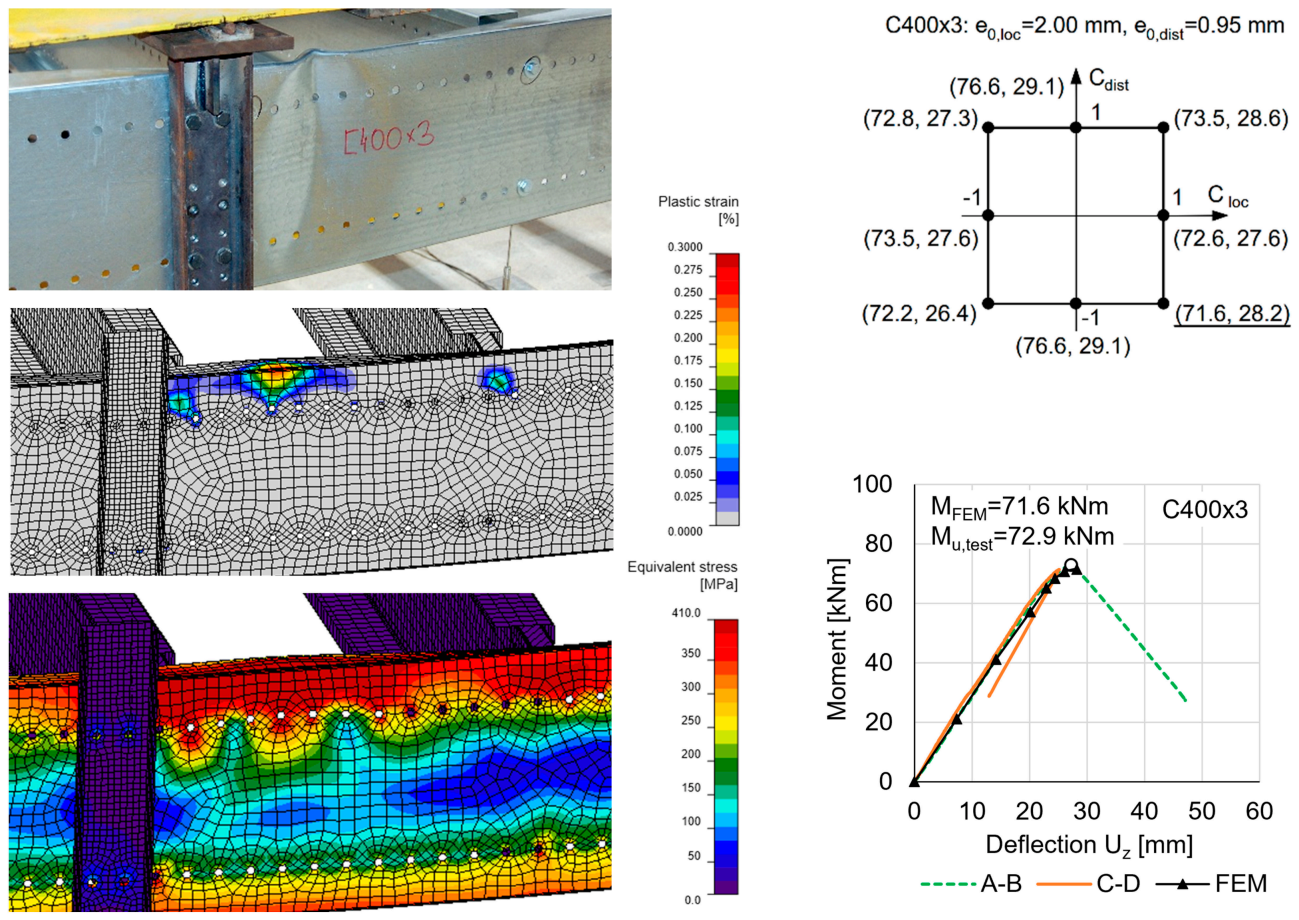
Experimental and numerical results for the C400 × 3 platform specimen.

**Figure 13:**
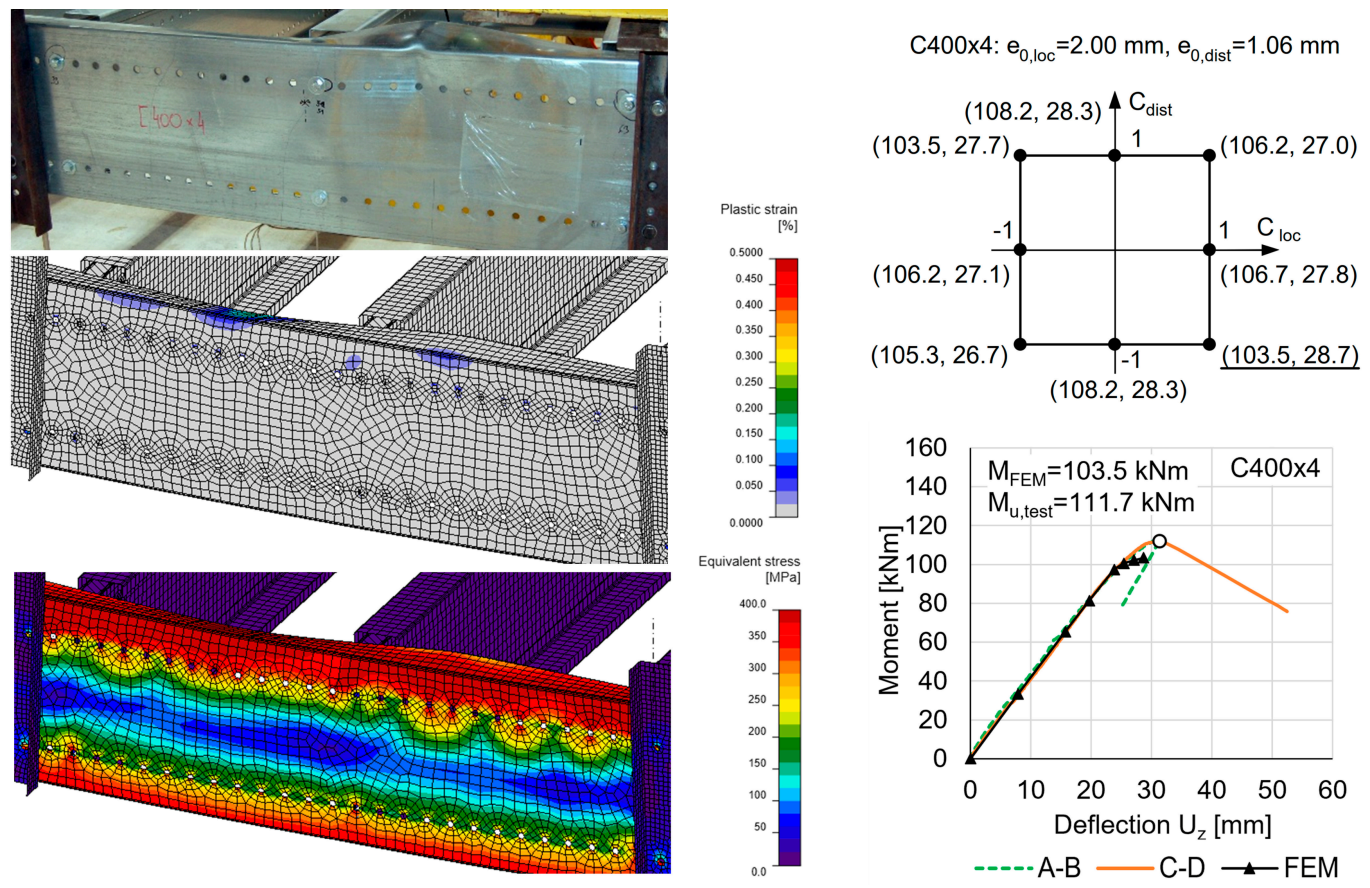
Experimental and numerical results for the C400 × 4 platform specimen.

**Figure 14:**
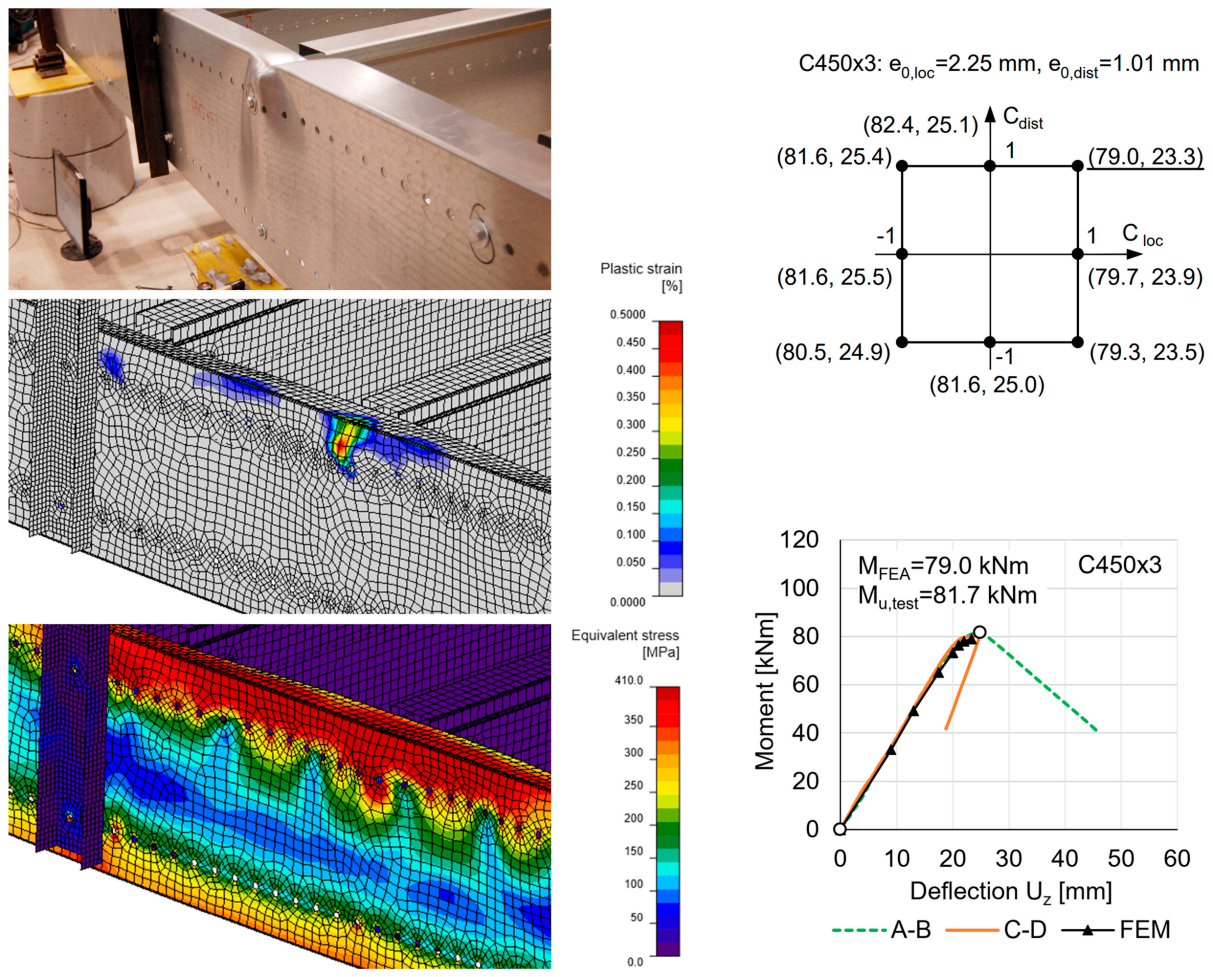
Experimental and numerical results for the C450 × 3 platform specimen.

**Figure 15:**
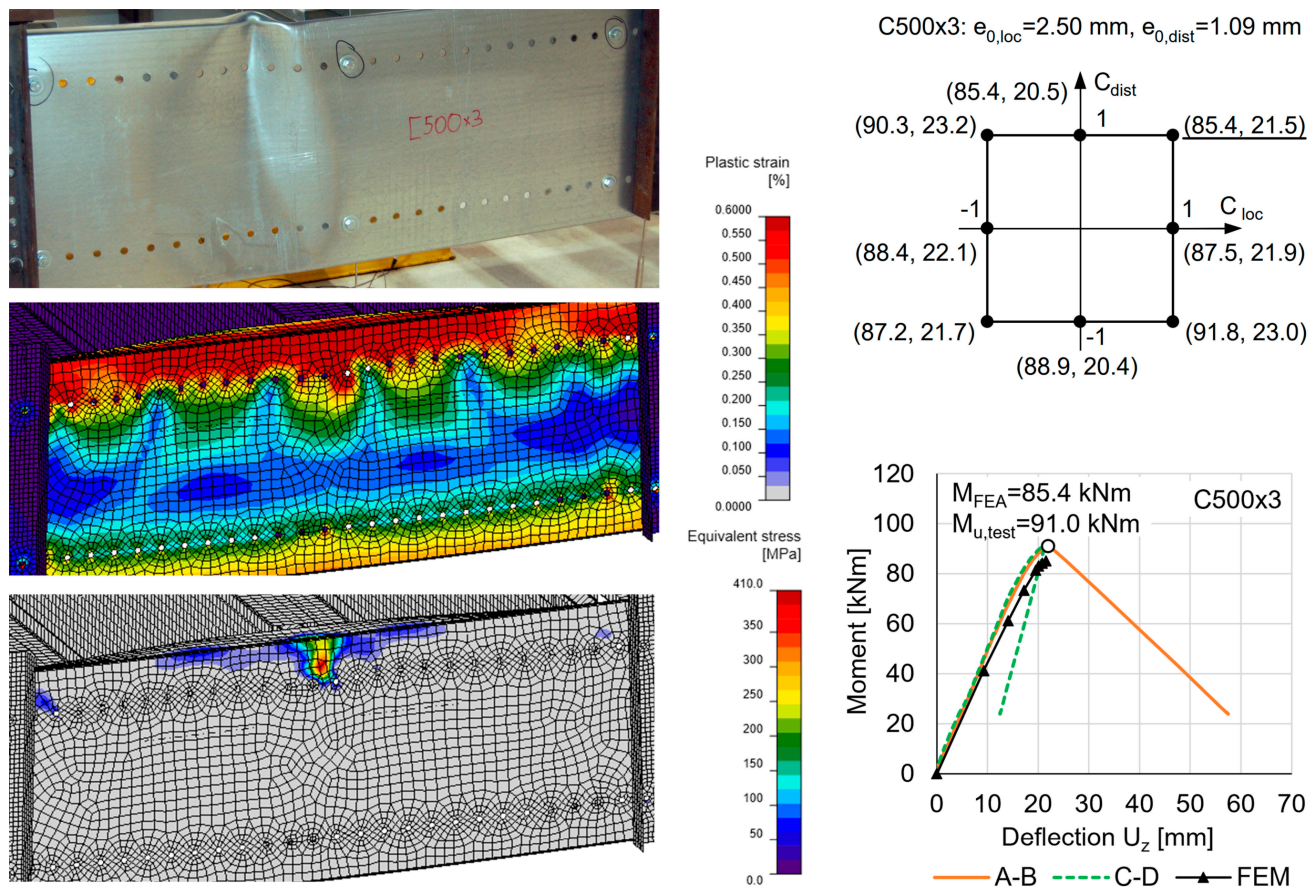
Experimental and numerical results for the C500 × 3 platform specimen.

**Figure 16:**
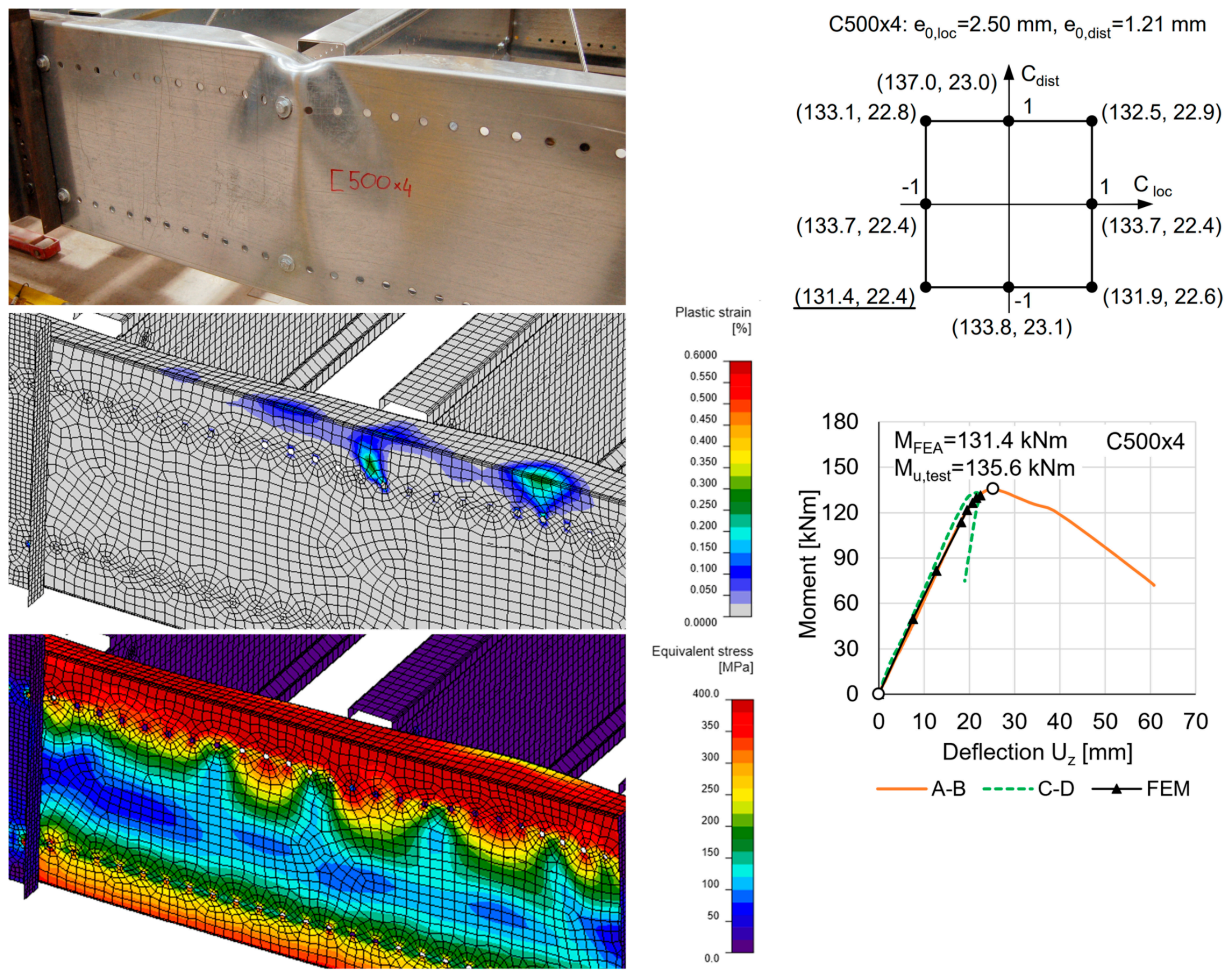
Experimental and numerical results for the C500 × 4 platform specimen.

At failure, the deformation pattern changed abruptly to the form shown in Figure 10, Figure 11, Figure 12, Figure 13, Figure 14, Figure 15 and Figure 16.

The resulting plastic hinge lines can be clearly observed in the photographs. The photographs are accompanied by contour plots of equivalent plastic strain and von Mises stress. In Figure 10, Figure 11, Figure 12, Figure 13, Figure 14, Figure 15 and Figure 16, the latter is labelled as Equivalent stress, following the terminology used in the software. Both contour plots correspond to the maximum load obtained in the GMNIA analyses. The maximum equivalent plastic strain did not exceed 0.6%, remaining well below the 5% limit specified in EN 1993-1-3 [7] for finite element analyses of cold-formed steel structures. The von Mises stress distributions indicate that the highest stresses developed in the compressed part of the web, particularly near the web-to-flange junction. The corresponding equivalent plastic strain distributions show that plastic deformation remained highly localized within this region, while the remaining parts of the cross-section exhibited only limited plasticity.

The results of the square-max analysis are shown in the upper-right corner of Figure 10, Figure 11, Figure 12, Figure 13, Figure 14, Figure 15 and Figure 16. The values given in parentheses represent the maximum bending moment, *M_FEM_* (kNm), and the corresponding deflection, *U_z,FEM_* (mm). When two imperfection combinations resulted in the same resistance, the case with the larger deflection was selected for further analysis. The finally adopted cases are underlined.

The last column of Table 4 presents the ratio of the effective section bending resistance calculated in accordance with EN 1993-1-3 [7] to the experimentally determined ultimate bending resistance. Despite neglecting the effect of web perforations, the effective cross-section approach according to [7] yielded predictions comparable in accuracy to the GMNIA analyses. This agreement is discussed further in Section 4.

**Figure 17:**
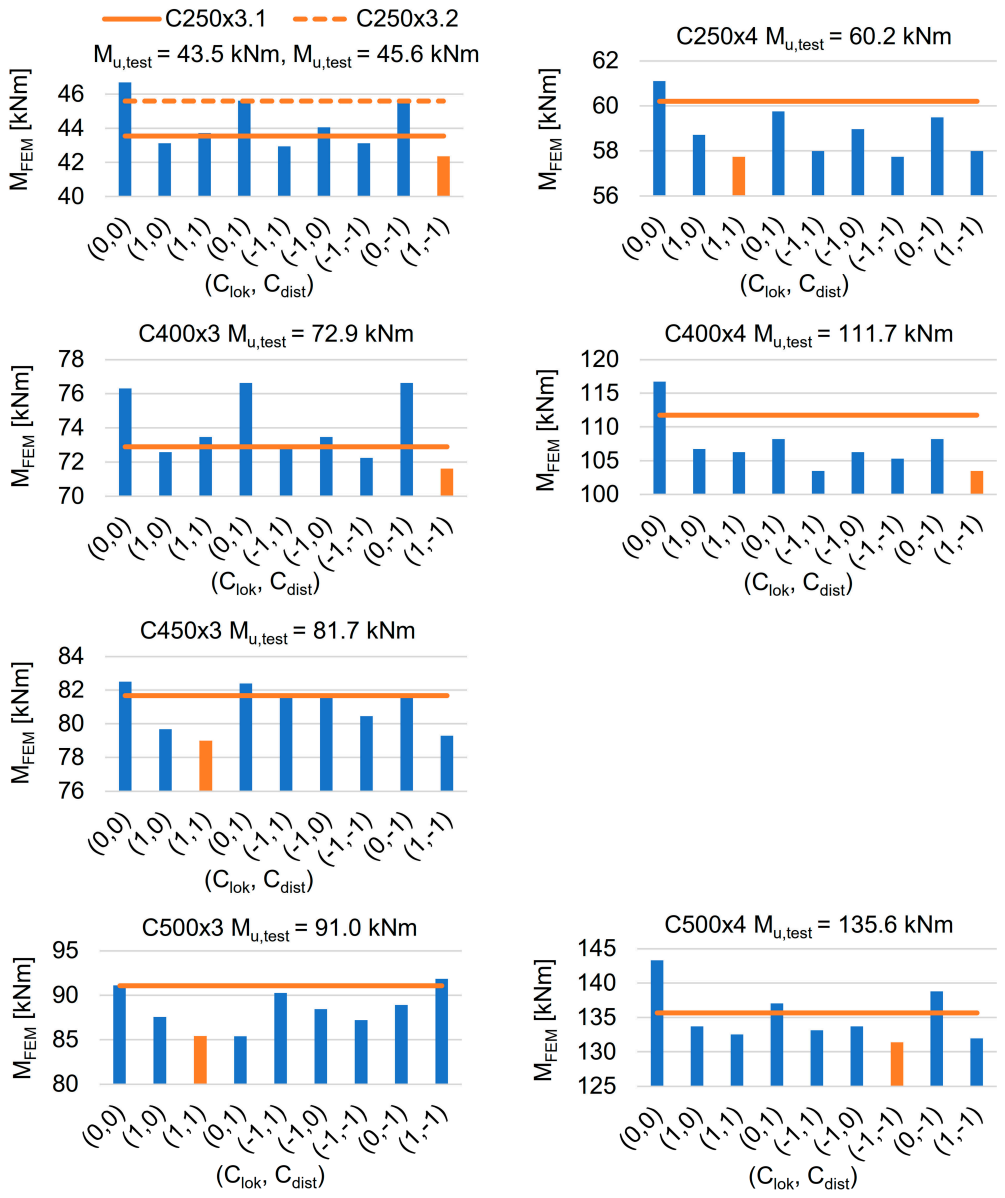
Results of the GMNIA sensitivity analysis of various combinations of local and distortional imperfections.

Figure 17 summarizes the ultimate resistance $M_{FEM}$ obtained for the eight geometric imperfection patterns illustrated in Figure 8a and represented in the upper-right corner of Figure 10, Figure 11, Figure 12, Figure 13, Figure 14, Figure 15 and Figure 16. For comparison, the results obtained for the initially perfect geometry ($C_{loc}=C_{dist}=0$) are also included. The horizontal lines represent the experimentally determined resistance $M_{u,test}$. The effect of the assumed imperfection pattern on the calculated resistance was relatively small, typically not exceeding a few percent. The difference between the minimum and maximum predicted resistance did not exceed approximately 6% for any specimen. Interestingly, the idealized model without imperfections did not always produce the highest resistance.

## 4. Discussion

### 4.1. Structural Response and Failure Mechanism

The moment–deflection curves shown in Figure 10, Figure 11, Figure 12, Figure 13, Figure 14, Figure 15 and Figure 16 illustrate the behaviour of the A–B and C–D main beams during the tests together with the corresponding FEM predictions. Analysis of these relationships identifies three distinct phases of structural response. In the first phase, extending up to approximately 0.5*M_u,test_*, the response is nearly linear-elastic. This is followed by a gradual reduction in stiffness associated with the development of local and distortional buckling modes (Phase II). This phase corresponds to the progressive growth of the local–distortional deformations shown in Figure 9. Beyond approximately 0.8*M_u,test_*, stiffness degradation accelerates markedly (Phase III), associated with the rapid development of plastic zones. In the beams that failed, the maximum load, *M_u,test_*, was followed by a sudden increase in permanent deflections as the structural system transformed into a mechanism. In contrast, the beams that did not lose their load-carrying capacity exhibited essentially elastic behaviour during unloading, although small residual deformations remained after completion of the loading cycle.

Overall, the observed deformation patterns indicate that the structural response was governed by progressive interaction between local and distortional buckling throughout the loading process rather than by a single dominant instability mode.

### 4.2. Accuracy of GMNIA Predictions

The GMNIA analyses reproduced the deformation patterns observed experimentally, confirming that the adopted modelling approach was capable of capturing the governing instability mechanisms. The predicted regions of maximum equivalent plastic strain corresponded closely to the experimentally observed locations of failure initiation, including the rotation of the compression flange, distortion of the flange-to-web junction and local web deformation. Neither the experiments nor the numerical analyses indicated that failure was governed by a single instability mode. Instead, distortional buckling initiated the loss of stiffness, followed by progressive local web yielding and plastic redistribution leading to the formation of the collapse mechanism. Throughout the loading process, distortional buckling and local yielding developed simultaneously and interacted, rather than acting as separate governing mechanisms.

The moment–deflection relationships obtained from the finite element analyses exhibited a similar three-stage response, in good agreement with the experimental observations. The analyses reproduced both the location of failure and the overall shape of the deformed members. The FEM model accurately predicted the ultimate resistance, yielding *M_FEM_*/*M_u,test_* ratios between 0.93 and 0.98. The corresponding deflections at peak load were also reproduced reasonably well.

The largest difference in the deformation response was observed for specimen C250 × 4, which was the stockiest of the investigated members. Although the ultimate moment was predicted with a difference of only 4.2%, the numerical model did not fully reproduce the gradual yielding and plastic redistribution observed experimentally. This discrepancy may partly result from the predefined bilinear material model available in Idea StatiCa and from the use of material properties obtained from coupons cut before press-braking. Consequently, the rounded stress–strain response and the local strength enhancement induced by cold forming in the bent regions were not explicitly represented. This interpretation remains tentative, as verification would require analyses using a more advanced material model.

### 4.3. Influence of Imperfection Combinations

The sensitivity study showed that the effect of the assumed combination of local and distortional imperfections on the predicted resistance was relatively small. For all investigated specimens, the difference between the minimum and maximum resistance obtained from the considered imperfection combinations did not exceed approximately 6%. Moreover, the model with initially perfect geometry did not always produce the highest resistance. This suggests that the ultimate resistance of the investigated structures was governed primarily by the overall instability mechanism rather than by the precise shape of the assumed geometric imperfections. Such behaviour is consistent with the relatively small spread of the critical load factors associated with the local and distortional modes identified in the LBA analyses.

### 4.4. Influence of Web Openings, Crossbeam Restraints and Applicability of EC3

The flexural behaviour and instability of cold-formed steel beams have been extensively investigated in previous decades, providing the basis for current design approaches. More recent studies have focused on the influence of web perforations on the flexural resistance and buckling behaviour of isolated channel beams [6,8,9]. Zhao et al. [8] demonstrated experimentally that web openings may fundamentally change the governing failure mechanism from pure local or distortional buckling to local–distortional interaction. Unlike the isolated members investigated in these studies, the present specimens form part of a complete storage-platform system.

Consequently, their behaviour was influenced not only by web perforations but also by the interaction between the main beams and the transverse members through the crossbeam-to-web connections. Compared with the reductions in flexural resistance reported for isolated perforated beams [9], the influence of web perforations appeared less pronounced, suggesting that the crossbeam-to-web connections may have partially compensated for their adverse effect.

A particularly interesting finding concerns the comparison with the effective section approach of EN 1993-1-3 [7]. Despite the presence of web perforations, which are not explicitly considered in the design procedure for channel sections, the calculated-to-tested resistance ratios ranged from 0.96 to 1.01. However, this agreement should be interpreted with caution. The relatively small, regularly spaced openings and the additional restraint provided by the angle-cleat connections may have modified the local boundary conditions of the web panels, thereby reducing the susceptibility to web deformation and distortional buckling.

The deformation patterns support this interpretation. In several specimens, the plastic folds remained confined to the web panels between adjacent angle cleats, whereas in specimens C450 × 3 and C500 × 4 they developed directly at the mid-span angle-cleat connection. This indicates that the connections not only limited the effective deformation length of the web but also introduced local stiffness discontinuities that influenced where deformation localized. Consequently, the structural response should be regarded as that of an interacting structural system rather than of isolated perforated beams. The favourable agreement between the Eurocode predictions and the experimental results is therefore likely attributable, at least in part, to a compensation between the detrimental influence of the perforations and the beneficial restraint provided by the crossbeam-to-web connections. Accordingly, the observed agreement should not be interpreted as evidence that the current Eurocode provisions fully capture the behaviour of perforated channel beams or can be extrapolated directly to members with different perforation patterns or connection arrangements.

The present observations are consistent with the findings of ref. [12], which showed that angle-cleat restraints modify the lateral-torsional buckling response of sigma-section beams. The present study indicates that similar restraints may also influence local and distortional buckling behaviour.

Further research should focus on quantifying the interaction between web perforations and the restraint provided by crossbeam-to-web connections. In particular, numerical parametric studies and dedicated experiments should investigate the influence of angle-cleat stiffness, crossbeam spacing and perforation geometry on local and distortional buckling resistance.

### 4.5. Limitations and Scope of Applicability

The numerical analyses intentionally adopted an engineering-oriented modelling strategy based on the recommendations of prEN 1993-1-14. Accordingly, several effects were represented in a simplified manner rather than modelled explicitly. Residual stresses were accounted for indirectly through equivalent geometric imperfections, whereas the material model was limited to the predefined bilinear constitutive relationship available in Idea StatiCa. Material properties were derived from coupons cut before press-braking, and therefore the local strength enhancement caused by cold forming was not considered explicitly. Although these simplifications did not prevent good agreement between the numerical predictions and the experimental resistance, their influence on the deformation response, particularly for stockier members, deserves further investigation.

Global bow imperfections were not included in the numerical analyses because neither the LBA results nor the experiments indicated susceptibility to global instability. The investigated platform system was effectively restrained against lateral displacement by the test arrangement, and no global buckling modes were identified among the first 30 eigenmodes. Consequently, the conclusions drawn in this study apply to structural systems with comparable restraint conditions. The influence of global imperfections may become more significant for isolated beams or structures with reduced lateral restraint.

In addition, with the exception of the C250 × 3 configuration, only one specimen was tested for each cross-section. Consequently, the specimen-to-specimen variability and the associated experimental uncertainty could not be quantified statistically. Although the good agreement between the experimental and numerical results supports the reliability of the observed trends, additional tests would be required to establish their statistical significance.

### 4.6. Additional Observations

Post-test inspection revealed permanent deformation of the bolt holes primarily in the connections between the main beams and the load transfer elements. In the most heavily loaded specimens, ovalization of the holes was observed in the webs of the main beams at the locations of the M14 bolts fitted in 14 mm diameter holes. The permanent elongation of the holes reached approximately 2 mm, indicating local bearing-induced plastic deformation of the web. In contrast, no noticeable tilting of the bolts, which typically precedes failure of single-shear bearing-type bolted connections, was observed. Similarly, no significant deformation of the holes in the load transfer elements or damage to the bolts was found. The bolted connections between the main beams and the crossbeams exhibited no significant permanent deformation. These observations indicate that although limited local slip associated with bearing deformation occurred in the load transfer element connections during loading, it did not govern the connection behaviour or the failure mechanism of the tested specimens. Therefore, the crossbeam-to-main beam connections remained structurally effective throughout the loading process, continuing to provide restraint to the webs of the main beams.

## 5. Conclusions

This paper presents the results of experimental and numerical investigations of the flexural behaviour of cold-formed steel storage-platform systems composed of perforated channel-section main beams and sigma-section crossbeams. Based on the present investigation, the following conclusions can be drawn:

- The failure mechanism of the investigated platforms was governed by the interaction between local and distortional buckling, accompanied by yielding in the compression zone. The GMNIA analyses accurately reproduced the observed deformation patterns, failure locations and ultimate resistance, with FEM-to-test resistance ratios ranging from 0.93 to 0.98.
- The assumed combination of local and distortional geometric imperfections had only a limited influence on the predicted resistance. For all investigated specimens, the difference between the minimum and maximum resistance obtained from the analysed imperfection combinations did not exceed approximately 6%, indicating that the predicted resistance was relatively insensitive to the particular imperfection combination adopted.
- For the investigated platform systems, the effective section approach according to EN 1993-1-3 provided accurate predictions of the ultimate resistance, with calculated-to-test resistance ratios ranging from 0.96 to 1.01.
- The experimental observations suggest that the interaction between the main beams and the transverse members through the crossbeam-to-web angle-cleat connections influenced the development of local and distortional deformations and may have partially compensated for the adverse effect of the web perforations. Consequently, the favourable agreement between the Eurocode predictions and the experimental results should not be interpreted as evidence that the current design provisions are generally applicable to perforated channel beams with different perforation patterns or connection arrangements.
- Further experimental and numerical studies are required to quantify the interaction between web perforations and crossbeam-to-web connections, particularly with respect to the influence of connection stiffness, crossbeam spacing and perforation geometry on local and distortional buckling resistance.

## Figures and Tables

**Table 1:** Nominal dimensions of the cross-sections (see Figure 2).

Main Beams/Crossbeams	t (mm)	h (mm)	w_c_ (mm)	w_s_ (mm)
C250 × 3/S250 × 3	3	250	150	110
C250 × 4/S250 × 4	4	250	150	110
C400 × 3/S400 × 3	3	400	240	200
C400 × 4/S400 × 4	4	400	240	200
C450 × 3/S450 × 3	3	450	280	240
C500 × 3/S500 × 3	3	500	320	280
C500 × 4/S500 × 4	4	500	320	280

**Table 2:** Results of the tensile coupon tests.

Coupon Thickness (mm)	Yield Stress (MPa)	Ultimate Stress (MPa)
3	410	482
4	400	506

**Table 3:** Maximum measured geometric imperfections.

Specimen	Global Bow (mm)	Web Crown *δ*_0,*cr(w)*_ (mm)	Flange Crown *δ*_0,*cr(f)*_ (mm)	Top Flange Flare *δ*_0,*f*_ (mm)
C250 × 3.1A–B	0.7	0.0	0.0	−0.1
C250 × 3.1C–D	0.5	0.1	0.0	−2.2
C250 × 3.2A–B	0.1	−0.2	0.0	−0.7
C250 × 3.2C–D	0.7	0.0	0.0	−0.3
C250 × 4A–B	0.2	−0.2	0.1	−1.6
C250 × 4C–D	0.3	0.0	0.1	−2.2
C400 × 3A–B	0.2	−0.2	−0.1	−1.1
C400 × 3C–D	0.0	0.2	0.1	−2.2
C400 × 4A–B	0.6	0.0	0.0	−4.2
C400 × 4C–D	0.3	0.1	−0.1	−2.0
C450 × 3A–B	1.0	0.1	0.1	−1.3
C450 × 3C–D	0.8	0.0	0.1	−1.7
C500 × 3A–B	0.9	0.0	−0.1	−2.4
C500 × 3C–D	0.2	0.1	0.0	−0.2
C500 × 4A–B	0.1	0.1	−0.1	−2.3
C500 × 4C–D	0.2	0.0	0.1	−2.2

**Table 4:** Summary of the test and FEM results.

Specimen	Test		FEM		Eurocode ^1^
	M_u,test_ (kNm)	U_z,test_ (mm)	M_FEM_/M_u,test_	U_z,FEM_/U_z,test_	W_eff_∙f_y_/M_u,test_
C250 × 3.1	43.5	47.0	0.97	0.89	1.01
C250 × 3.2	45.6	52.1	0.93	0.81	0.96
C250 × 4	60.2	60.2	0.96	0.68	0.98
C400 × 3	72.9	27.3	0.98	1.03	1.01
C400 × 4	111.7	31.4	0.93	0.91	0.96
C450 × 3	81.7	24.8	0.97	0.94	1.00
C500 × 3	91.0	22.0	0.94	0.98	1.00
C500 × 4	135.6	25.2	0.97	0.89	1.00

^1^ effective cross-section modulus according to EN1993-1-3 [7].

## Data Availability

The original contributions presented in this study are included in the article. Further inquiries can be directed to the corresponding author.

## Abbreviations

The following abbreviations are used in this manuscript:

CBFEM	Component-Based Finite Element Method
FE	Finite Element
CFS	Cold-Formed Steel
FEM	Finite Element Method
GMNIA	Geometrically and Materially Nonlinear Analysis with Imperfections
LBA	Linear Buckling Analysis
LVDT	Linear Variable Differential Transformer
MNA	Materially Nonlinear Analysis
